# Contrast-enhanced ultrasound (CEUS) in patients with metastasis-like hepatic alveolar echinococcosis: a cohort study

**DOI:** 10.1007/s40477-022-00688-x

**Published:** 2022-05-21

**Authors:** Melissa Schweizer, Julian Schmidberger, Patrycja Schlingeloff, Wolfgang Kratzer

**Affiliations:** Department of Internal Medicine I, University Hospital Ulm, Albert-Einstein-Allee 23, 89081 Ulm, Germany

**Keywords:** Hepatic Alveolar Echinococcosis (HAE), Contrast-Enhanced Ultrasound (CEUS), Ultrasonography (US), Liver, Diagnostic

## Abstract

**Purpose:**

Hepatic alveolar echinococcosis (HAE) of the metastasis-like pattern, according to the Echinococcus Ulm classification, is usually discovered as an incidental finding, and the diagnostic differentiation from “true metastases” is difficult. The aim of this study was to investigate whether lesions of the “metastasis-like pattern” in HAE show a typical contrast behavior that can be used for differentiation from metastasis in malignancies.

**Methods:**

This prospective clinical study included 11 patients with histologically confirmed HAE of the metastasis-like pattern (7 female and 4 male; mean age, 57.1 years; mean disease duration, 59.5 months), who had been examined by B-scan sonography and CEUS, from the National Echinococcosis Registry Germany.

**Results:**

On contrast-enhanced sonography, 11/11 reference lesions showed annular rim enhancement in the arterial and portal venous phases. Throughout the entire 4-min study period, none of the reference lesions showed central contrast enhancement—i.e., all exhibited a complete “black hole sign”. A small central scar was seen in 81.8% of cases.

**Conclusion:**

In clinically unremarkable patients with incidentally detected metastasis-like lesions of the liver, contrast-enhanced sonographic detection of rim enhancement without central contrast uptake (black hole sign) should be considered evidence supporting a diagnosis of hepatic alveolar echinococcosis with a rare metastasis-like pattern. This can help to differentiate HAE from metastases, especially in high-endemic areas.

## Introduction

Alveolar and cystic echinococcosis are two completely different diseases [1]. Cystic echinococcosis (CE) usually presents sonographically as cystic lesions and often have an easier to differentiate pattern [2]. The sonographic findings in hepatic alveolar echinococcosis (HAE) are complex and can pose significant differential diagnostic problems. They may present like complicated cysts or malignant tumors in the liver [2]. HAE is a rare disease, with only approximately 18,000 new cases per year worldwide. The parasite is endemic in Germany, France, Austria, and Switzerland, as well as Central Asia and Western China thus predominantly in the Northern Hemisphere, while cystic echinococcosis occurs mainly in the Southern Hemisphere [3]. HAE starts with a long initial asymptomatic period [4]. The exact incubation period of the parasite is unknown, but is estimated to be 5–15 years. In over 98% of cases, the liver is the most frequently affected organ [1].

**Fig. 1:**
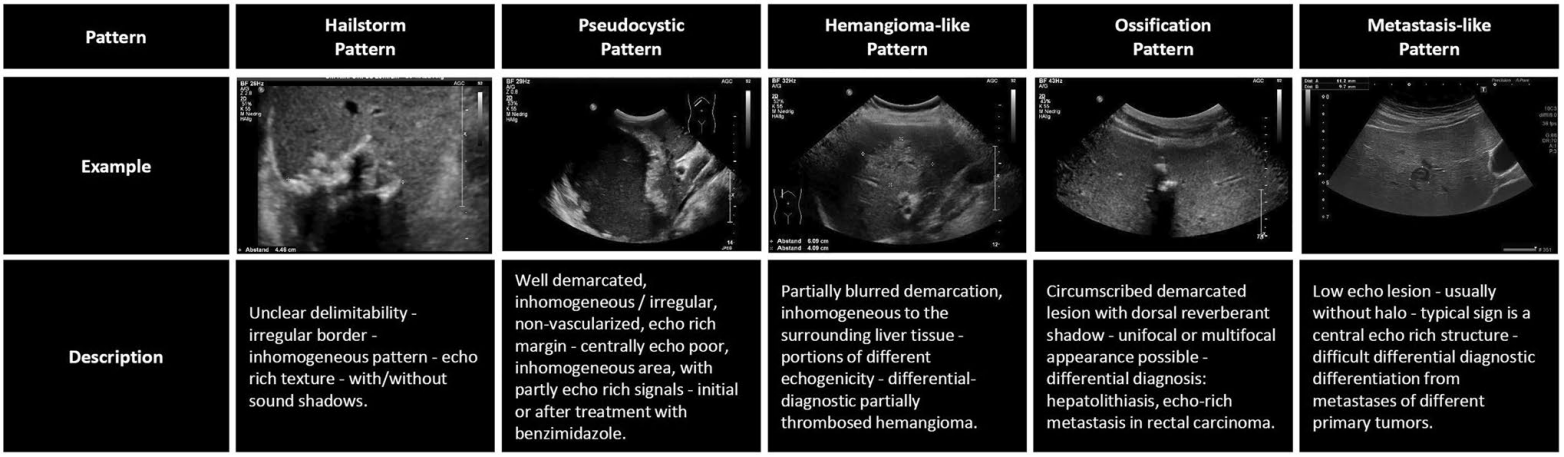
Illustration of the different B-scan sonographic patterns according to the *Echinococcus multilocularis* Ulm (EMUC-US) classification. Source: Kratzer W, Weimer H, Schmidberger. Echinokokkose: eine Herausforderung der Lebersonographie. Ultraschall in Med 2021. https://doi.org/10.1055/a-1694-5552

**Fig. 2:**
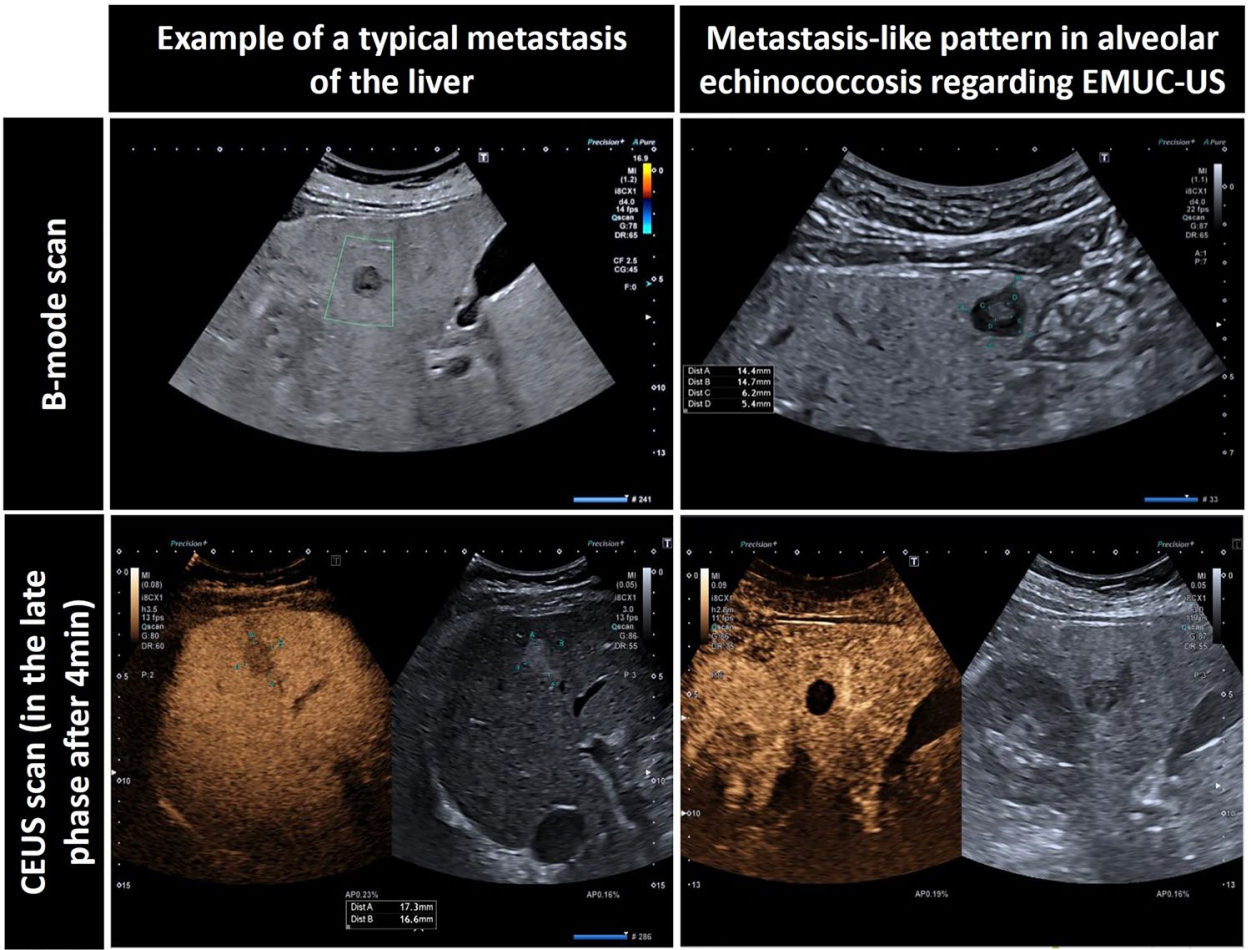
The figure shows a typical liver metastasis as well as a metastasis-like pattern in alveolar echinococcosis according to EMUC-US in the B-scan as well as the corresponding contrast behavior in the late phase after 4 min in contrast-enhanced ultrasound (CEUS). Source: Kratzer W, Weimer H, Schmidberger. Echinokokkose: eine Herausforderung der Lebersonographie. Ultraschall in Med 2021. https://doi.org/10.1055/a-1694-5552

In hepatic alveolar echinococcosis (HAE), the parasitic lesions show highly diverse sonomorphological presentations, and are often difficult to diagnose by B-scan sonography [5, 6]. In particular, differentiation from "true" metastases and cholangiocellular carcinomas (CCC) can pose a significant differential diagnostic problem [7, 8]. The *Echinococcus multilocularis* Ulm Classification Ultrasound (EMUC-US) is the first method enabling classification into five B-scan sonographic patterns: hailstorm, pseudocystic, ossification, hemangioma-like, and metastasis-like (Fig. 1) [6]. However, B-scan ultrasonography cannot reliably distinguish between an AE lesion of the metastasis-like pattern versus a "true" hepatic metastasis of another primary tumor (Fig. 2). Therefore, suspicion of liver metastasis often leads to extensive diagnostic staging [7–11].

In addition to US examination for the detection of HAE, imaging diagnosis is usually complemented by CT scan which best shows common HAE characteristic calcifications. Furthermore, HAE-related morphologic changes are comprehensively visualized by CT [12].

Furthermore, positron emission computed tomography (PET-CT) is of crucial importance. It is the only imaging technique that allows an assessment of inflammatory activity of the disease based on metabolic activity. FDG accumulation, usually in the marginal area of a lesion, is a parameter for the metabolic activity of the parasite. This is particularly helpful in the assessment of progression under benzimidazole (BMZ) therapy and in the detection of recurrences, e.g., in the postoperative course [13].

Since the introduction of second-generation ultrasound contrast enhancers, contrast-enhanced ultrasound (CEUS) has become the standard method of liver metastases diagnosis, due to improved imaging in the portal venous and late phases [14]. Meanwhile, the distinction between hyper-vascularized and hypo-vascularized metastases has been established. In most cases, typical metastases show hypo-enhancement in the portal venous phase, while both hypo-enhancement and non-enhancement can be observed in the late phase [14]. Typical contrast behavior, with hyper-enhancement in the arterial phase and subsequent "washout" and hypo-enhancement in the late phase, has also been observed in small metastases with a diameter of < 20 mm. Small metastases are particularly likely to show hyper-enhancement since they have barely any necrotic areas [15].

In recent years, CEUS has become increasingly important in the diagnosis of HAE [16–23]. Importantly, misdiagnosis of HAE in unclear metastatic lesions can lead to inappropriate treatment strategies and significantly delayed diagnosis [8]. A recent CEUS study in a rat model allowed imaging in the early stages of alveolar echinococcosis infection, and revealed that 28 of 30 lesions exhibited annular rim enhancement in the arterial phase, which corresponded to histological findings of inflammatory rim reaction [21].

The aim of this study was to investigate whether lesions of the “metastasis-like pattern type” in HAE show a typical contrast behavior that can be used for differentiation from metastasis in malignancies.

## Material and methods

### Patients

A total of 11 patients with histologically confirmed HAE and proven metastasis-like pattern, according to the EMUC-US classification, identified from the National Echinococcosis Registry Germany, were contacted and consented for prospective participation in the study [6, 24]. All patients were examined by B-scan ultrasound and CEUS (Table 1). At baseline, B-scan ultrasonography was performed to determine the number of metastatic lesions. The largest metastasis-like lesion was selected as the reference lesion, and B-scan sonography was used to assess its location, size, shape, and echogenicity. Before CEUS was performed, all reference lesions were examined for detectable vascularization by color-coded duplex ultrasonography, power Doppler, and superb microvascular imaging (SMI) [25]. All patients gave their written informed consent before inclusion in the study. The study was conducted in accordance with the Declaration of Helsinki and was approved by the local ethics committees (23/20) [26].

**Table 1:** Characteristics of the analyzed patients with metastasis-like pattern in alveolar echinococcosis according to EMUC-US

Total (*n* = 11)	Frequency (%)
Age, years	
Mean ± SD (median)	57.09 ± 15.33 (65.00)
Min–max	32.00–76.00
Gender	
Male	4 (36.36%)
Female	7 (63.64%)
BMI, kg/m^2^	
BMI < 25	2 (18.18%)
BMI 25–30	7 (63.64%)
BMI ≥ 30	2 (18.18%)
Disease duration, months	
Mean ± SD (median)	59.45 ± 46.19 (43.00)
Min–Max	5.00–166.00
Suspected diagnosis	
Echinococcosis	2 (18.18%)
Suspicion of malignancy of unclear genesis	8 (72.73%)
Other suspected diagnoses	1 (9.09%)
Incidental finding	
Yes	11 (100.00%)
No	0 (0.00%)
Benzimidazole therapy	
Yes	9 (81.82%)
No	1 (9.09%)
With delay	1 (9.09%)
Benzimidazole duration	
Mean ± SD (median)	38.45 ± 48.42 (26.00)
Min–Max	0–166.00
Benzimidazole toxicity	
Yes	4 (36.36%)
No	7 (63.64%)

### Contrast-enhanced ultrasound (CEUS)

Contrast-enhanced ultrasound was performed following the guidelines and good clinical practice of the EFSUMB [27]. All examinations were performed using an Aplio I800 (Canon Medical Systems, Tochigi, Japan), with a C1-5 MHz convex transducer, SonoVue (Bracco Medical Imaging Germany Ltd., Konstanz, Germany) was used as contrast medium. The investigator gave each patient individual instructions for inspiration and expiration. In general, 1.2 ml of SonoVue was injected followed by 10 ml of 0.9% NaCl; for subcapsular tumors, 1.6 ml of SonoVue.

The period of 7–30 s post-injection (p.i.) was defined as the arterial phase, 31–120 s p.i. as the portal phase, and 121–360 s p.i. as the late phase. Video recordings were obtained during inflation of the contrast agent (contrast arrival time) and the arterial phase (7–30 s p.i.). A targeted punctate scanning of the liver (sweep) was performed in the portal venous phase (60 s p.i.), and additional sweeps were performed in the venous phase starting at 120 and 180 s, respectively. The total observation time was 4 min.

### Statistical analysis

Statistical analysis was performed using SAS version 9.4. We calculated frequencies, mean, and location and dispersion measures. Differences were determined using the nonparametric Mann-Whitney *U* test. The tests were two-sided. A *p* value of < 0.05 (*α* = 0.05) was considered statistically significant with a 5% probability of error.

## Results

In our cohort of 11 patients with alveolar echinococcosis and sonomorphological metastasis-like EMUC-US pattern, the gender distribution was 63.6% female (7/11) to 36.4% male (4/11). The mean patient age at the time of the study was 57.1 ± 15.3 years. In 7/11 (63.6%) of the patients, the BMI was 25–30. In 2/11 (18.2%) patients, echinococcosis was initially suspected, in 8/11 (72.7%), suspicion of malignancy of unclear genesis, and in one patient, other suspected diagnoses were assumed. The mean disease duration in patients with alveolar echinococcosis was 59.5 ± 46.2 months. A total of 9/11 (81.8%) patients received a benzimidazole therapy, one patient did not receive BMZ therapy and one patient received an outlet attempt. The average BMZ therapy duration was 38.5 ± 48.4 months. Benzimidazole toxicity occurred in 4/11 (36,4%) during BMZ therapy (Table 1). The male and female patients did not significantly differ in BMI (*p* = 0.7048), age (*p* = 0.5074), disease duration (*p* = 0.2193), or duration of benzimidazole therapy (*p* = 0.1564).

**Fig. 3:**
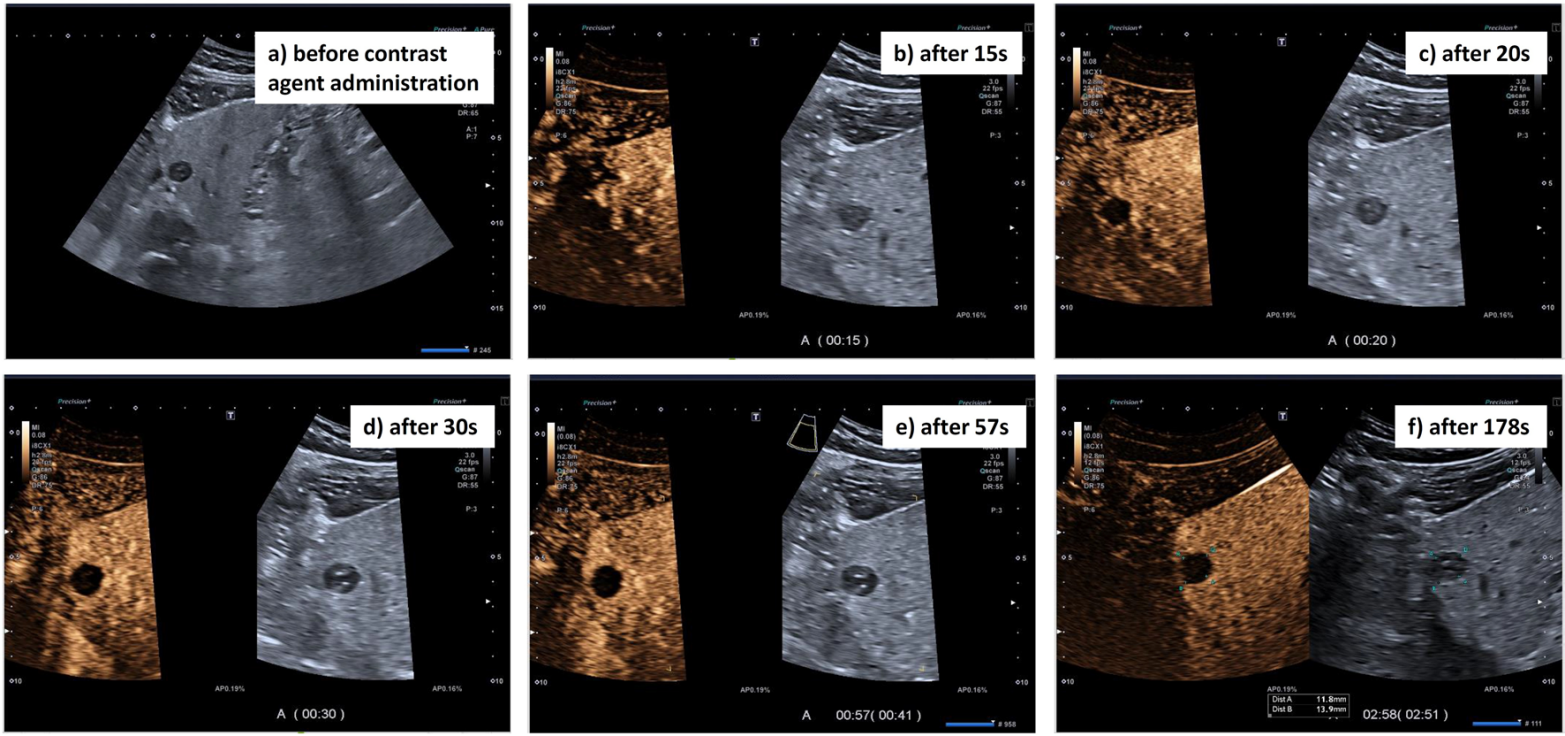
Case series examining the contrast response of alveolar echinococcosis (AE) lesions with a metastasis-like pattern. **a** Overview of the liver on B-scan. **b–f** Contrast flooding after 15 s (**b**), 20 s (**c**), 30 s (**d**), 57 s (**e**), and 178 s (**f**)

Before inclusion of patients in the National Echinococcosis Registry Germany, in 100% of patients (11/11), AE was discovered as an incidental finding during the investigation of other non-hepatic complaints or during screening examinations. A total of 10 of the lesions were biopsied and one patient underwent surgery. In each case, the entire liver showed a metastasis-like pattern of AE, with a mean of 3.6 ± 2.4 lesions (range: 1–10). The majority of the reference lesions (9/11) were located in the left lobe of the liver (Fig. 3a). Specifically, the reference lesion was most frequently located in liver segment II (27.3%, 3/11). The mean reference lesion size was 15.7 ± 6.4 mm (range: 8.0–32.0 mm). The reference lesion shape was round or oval in 45.4% (5/11), and polycyclic in 9.1% (1/11). All reference lesions were sharply demarcated, low in echo, and homogeneous (100%, 11/11). A small central echoic scar was visible in 81.8% (9/11) of the reference lesions (Table 2).

**Table 2:** Description of the lesion on contrast-enhanced ultrasound (CEUS)

Total (*n* = 11)	Frequency (%)
SonoVue	
Mean ± SD (Median)	1.29 ± 0.16 (1.20)
Min–max	1.20–1.60
Arrival time	
Mean ± SD (Median)	11.09 ± 2.66 (11.00)
Min–max	7.00–16.00
Central contrast agent acquisition	
No	11 (100.00%)
Yes	0 (0.00%)
Rim enhancement	
No	0 (0.00%)
Yes	11 (100.00%)
Number of lesions	
Mean ± SD (median)	3.64 ± 2.42 (3.00)
Min–Max	1.00–10.00
Reference lesion location, segment	
Segment I	0 (0.00%)
Segment II	3 (27.27%)
Segment III	2 (18.18%)
Segment IVa	2 (18.18%)
Segment IVb	2 (18.18%)
Segment V	1 (9.09%)
Segment VI	0 (0.00%)
Segment VII	0 (0.00%)
Segment VIII	1 (9.09%)
Lesion size, mm	
Mean ± SD (median)	15.73 ± 6.40 (15.00)
Min–Max	8.00–32.00

None of the lesions (0/11) showed a signal with any of the conventional Doppler techniques. All lesions (100%, 11/11) exhibited annular rim enhancement in the arterial and portal venous phases (Fig. 3b–f). The amount of SonoVue administered was 1.2 ml in 8/11 cases, 1.6 ml in 2/11 cases, and 1.4 ml in 1/11 cases. The mean contrast agent arrival time was 11.1 ± 2.7 s. None of the lesions (0/11) showed central contrast uptake over a period of 4 min (Table 2).

## Discussion

This is the first prospective study to characterize the contrast pattern of metastatic liver lesions according to the EMUC-US classification in HAE [3]. Prior to inclusion in the National Echinococcosis Registry Germany [24], the metastatic lesion was discovered incidentally in all patients. None of the patients showed findings for an underlying malignant disease. This initially led to the diagnosis of “malignancy of unclear etiology” in 73% of cases.

The current literature confirms that HAE is commonly misdiagnosed as liver metastases [7, 8, 28, 29]. The differential diagnosis of metastasis-like AE is complicated by the fact that it is a rare morphology of AE, comprising only 6.5% of HAE cases [6]. In our study, the majority of the reference lesions were located in the left liver lobe. In other studies, the majority of lesions were mostly described in the right liver lobe. Most frequently, the reference lesion was localized in segment 2 of the liver (~ 27%), in other studies only in 10.3% [6, 22]. None of the metastasis-specific lesions had a halo sign on B-scan ultrasonography, which is considered a malignancy criterion for identifying “true” metastases [16].

On contrast-enhanced ultrasound, all 11 reference lesions exhibited annular rim enhancement in the arterial phase. The onset of this phenomenon coincided with the arrival time of the contrast enhancer at 11 s post-injection. The rim enhancement disappeared towards the end of the portal venous phase (> 60 s after injection). Tao et al. first described rim enhancement as a band-like enhancement in the arterial phase around the irregular rim of the lesion. This report also described the phenomenon of the “black hole”, a complete non-enhancement of the interior of the AE lesion [20]. This observation of a late washout of rim enhancement in HAE lesions was also reported by Wa et al., who highlighted it as a key criterion for the differential diagnosis of intrahepatic cholangiocellular carcinoma (CCC) [11]. In a retrospective study regarding the form of rim enhancement, Cai et al. examined 43 reference lesions and reported that 11 exhibited “ring-like” hyper-enhancement in the arterial phase [29]. Similarly, Li et al. examined 39 AE lesions, and found that 4 exhibited annular enhancement, which rapidly developed in the arterial phase and then slowly subsided in the portal venous or late phase [30]. Neither of those studies determined the B-scan morphology of the AE lesions according to EMUC-US; therefore, no conclusions can be drawn regarding the extent to which annular rim enhancement may have corresponded to a metastasis-like pattern. A recent CEUS study in a rat model allowed imaging in the early stages of alveolar echinococcosis infection, and revealed that 28 of 30 lesions exhibited annular rim enhancement in the arterial phase, which corresponded to histological findings of inflammatory rim reaction [21]. In line with this study, Grimm et al. confirmed in a retrospective comparative study between histology and CT in HAE biopsies that no vessels could be detected in type IV lesions (early manifestation of HAE) considering the CT-specific echinococcal classification (EMUC–CT) [31, 32]. In contrast, in a comparative study between histology and CEUS, in different metastases, we were able to detect vascularization in metastases by immunohistochemistry using the vascular specific antibody CD34 [25]. Two additional observations support this hypothesis that circular rim enhancement is specific for the metastasis-like pattern. First, larger lesions exhibited irregular band-like enhancement and, second, rim enhancement was also observed in a study that explicitly examined small hemangioma-like and metastasis-like AE lesions [28, 29]. The AE lesions in our study showed non-enhancement in the late phase. This phenomenon has already been discussed by Cai et al., who performed a retrospective study including comparison of CEUS results with the histopathological sections. Our present findings confirm this assumption. The non-enhancement of metastasis-like AE lesions may support their differentiation from metastasis and CCC, which typically shows hypo-enhancement in the late phase. This finding is confirmed by two recent studies on the differentiation between hepatocellular carcinoma (HCC) and CCC. Both HCC and CCC show a wash-out phenomenon which is not sufficient to differentiate between HCC and CCC [33, 34]. In contrast none of the HAE lesions of metastasis-like pattern examined showed in our study exhibited central contrast uptake. This is a main criterion for distinguishing a metastasis-like AE lesion from a metastasis, since metastases exhibit at least slight enhancement in the arterial phase [16]. This is of particular clinical importance because in a large number of patients with HAE, the differential diagnosis of CCC is often initially discussed and patients are often unnecessarily initially faced with a malignant cancer diagnosis that is psychologically very stressful [8]. Since the mean diameter of lesions in our study was 15 mm, our results are particularly relevant to the delineation of small metastases. In such cases, delineation is particularly simple, as small metastases (< 20 mm) show complete hyper-enhancement in 43.9% of cases. Even hypo-vascularized metastases of < 20 mm in size can take up contrast medium centrally in the arterial phase, as necrosis areas are often not yet formed [35]. The present study has several limitations. The metastasis-like pattern, according to EMUC-US Ulm classification, is very rare. Therefore, our study included only 11 patients despite the recruitment of patients from the National Echinococcosis Registry Germany. Another limitation is the lack of a prospective comparison cohort with “real” metastases.

## Conclusions

In conclusion, our results showed that when contrast-enhanced sonography is performed in cases of HAE of the metastasis-like type, the typical findings are echo-poor small metastasis-like lesions with annular rim enhancement in the arterial phase, a “black hole sign” and a small central echoic scar. This information may help in making the difficult diagnosis of HAE in asymptomatic patients, especially in high-endemic areas.
